# Case Report: Clinical phenotypes and recombinant human growth hormone therapeutic exploration for a patient with Takenouchi-Kosaki syndrome harboring the *CDC42* p.Arg68Gln variant

**DOI:** 10.3389/fped.2026.1906125

**Published:** 2026-09-10

**Authors:** Xiao-xiao Lin, Yuan-yuan Song, Jia-cheng Xue, Hua Liu

**Affiliations:** 1 Department of Pediatrics, The First Clinical Medical College of Guangzhou University of Chinese Medicine, Guangzhou, Guangdong, China; 2 Department of Pediatrics, Guangzhou University of Chinese Medicine First Affiliated Hospital, Guangzhou, Guangdong, China

**Keywords:** case report, hearing loss, recombinant human growth hormone, short stature, Takenouchi-Kosaki syndrome

## Abstract

This study reports a pediatric patient harboring the heterozygous *CDC42* variant (c. 203G > A, p. Arg68Gln), an ultra-rare variant with limited clinical and genetic data worldwide. She presented with typical Takenouchi-Kosaki syndrome (TKS) manifestations, including severe growth retardation, characteristic craniofacial dysmorphism, persistent macrothrombocytopenia and progressive sensorineural hearing loss. Additional evaluations identified leukopenia, hypogammaglobulinemia and a humoral immunodeficiency. Compound heterozygous *GJB2* variants were detected but unlikely to dominate the patient's profound hearing loss. Off-label recombinant human growth hormone (rhGH) therapy was administered for her short stature. During six months of follow-up, favorable catch-up growth was achieved. Literature review was conducted to clarify features of this variant. This study supplements the clinical data of the *CDC42* p. Arg68Gln variant, provides preliminary evidence for rhGH intervention, and offers reference information for the clinical management and genetic counseling of patients with TKS.

## Introduction

Takenouchi-Kosaki syndrome (TKS) is a rare congenital multisystem developmental disorder caused by pathogenic variants in *CDC42*, clinically characterized by macrothrombocytopenia, distinctive craniofacial dysmorphism, growth retardation, lymphatic abnormalities, and variable neurodevelopmental delays (1). *CDC42* encodes a core small GTPase of the Rho family that serves as a pivotal molecular switch, dynamically cycling between GTP-bound active and GDP-bound inactive conformations and interacting with over 45 downstream effector proteins to precisely regulate fundamental cellular events, including proliferation, differentiation, cytoskeletal remodeling, migration, and apoptosis (2–4). The functional homeostasis of *CDC42* is essential for embryonic tissue and organ morphogenesis and sustains the development and functional maturation of the nervous, hematopoietic, and craniofacial systems throughout embryonic and postnatal life (5–7). Consequently, pathogenic *CDC42* variants result in a broad spectrum of TKS phenotypes.

Among the reported TKS cases, short stature is nearly universal across affected individuals. However, no interventional study targeting growth failure in TKS has been reported to date. This study describes a newly identified TKS patient harboring the rare *CDC42* p. Arg68Gln variant who received rhGH intervention for short stature. Our findings expand the phenotypic spectrum of this variant and provide preliminary real-world evidence to support further investigation into therapies for TKS-related growth failure.

## Method

All clinical laboratory and imaging data of the patient were obtained at our hospital. Genetic analysis was performed to identify the pathogenic variant by KingMed. A review of articles was conducted using the PubMed, Google Scholar, and Mendeley search engines. The keywords used were “Takenouchi-Kosaki syndrome”, “*CDC42*” and “*GJB2*”. From the retrieved articles, those relevant to the topic of this study and valuable as sources of information were selected.

## Case presentation

The patient was born at term by cesarean section and her mother developed gestational hypertension during pregnancy. She had a birth weight of 2,500 g and a birth length of 50 cm, and was diagnosed with congenital atrial septal defect and sensorineural hearing loss (SNHL). She presented with significant developmental delay, including crawling at 1 year of age and independent walking at 2. 5 years of age, accompanied by impaired academic performance and motor skills. Her elder sister and brother had normal growth and development consistent with typical peers. The paternal height was 169 cm and the maternal height was 156 cm. The family had no relevant hereditary diseases, chronic illnesses, or history of malignancy. Cochlear implantation was performed at the age of 6 years and 10 months.

**Figure 1:**
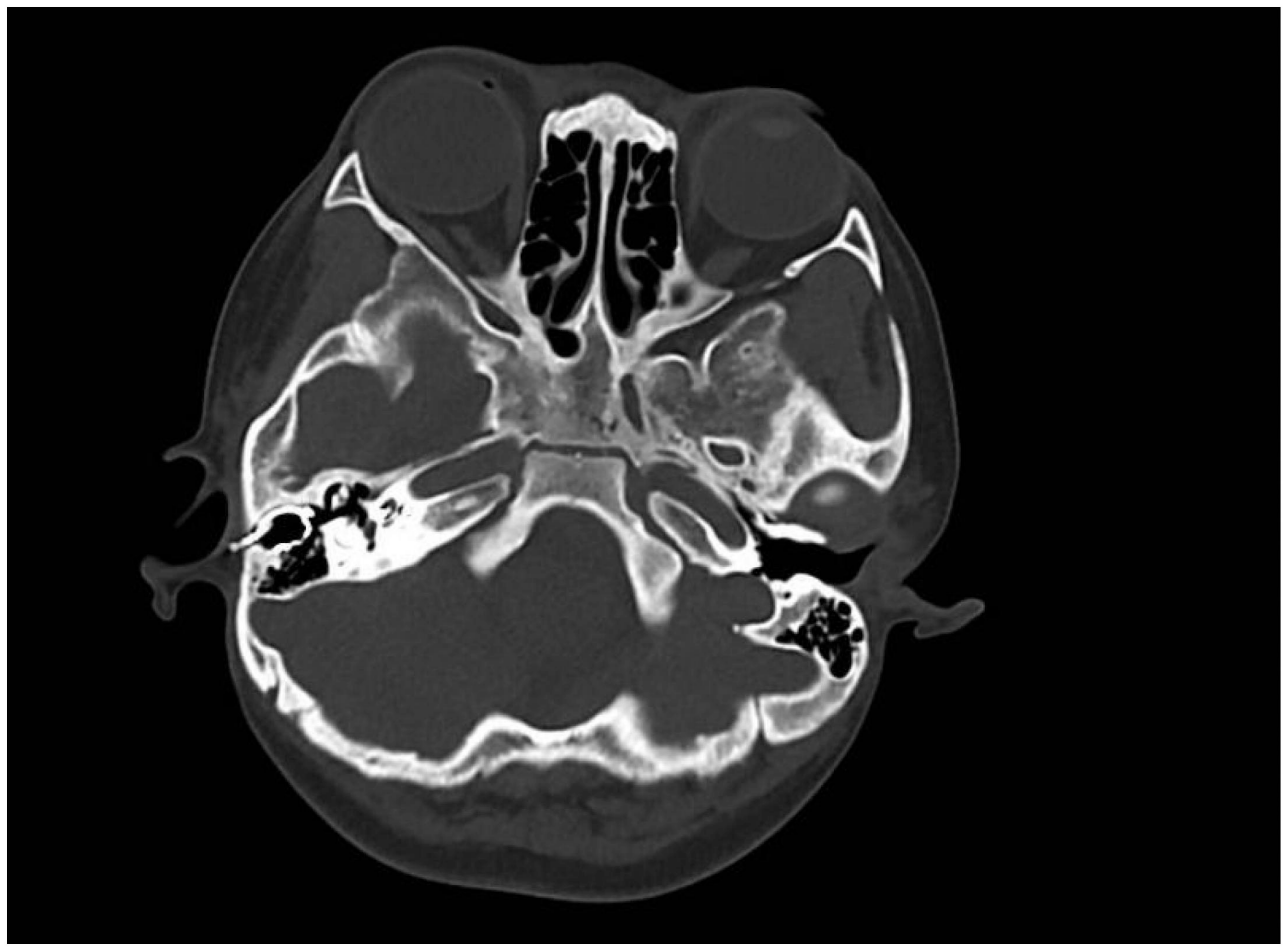
Brain MRI image of the patient.

The patient presented to the pediatric department at 11 years of age due to short stature, with a height of 123. 7 cm (−4. 35 SD, according to the 2005 growth reference for Chinese children established by the Capital Institute of Pediatrics). Her annual growth rate was unknown. Physical examination revealed characteristic facial dysmorphic features, including hypertelorism, depressed nasal bridge, widened nasal alar, high-arched palate, micrognathia, malocclusion, and thick lips, as well as low-set ears, slender fingers and camptodactyly. No palpable lymphadenopathy was noted. Laboratory tests showed a reduced IGF-1 level of 174. 7 ng/mL (−2. 0 SD, according to age- and sex-specific IGF-1 reference intervals established by KingMed Diagnostics). Routine blood tests indicated a reduced platelet count of 118 × 10⁹/L (167–453 × 10⁹/L) accompanied by a mildly elevated platelet-large cell ratio (P-LCR). The International Society on Thrombosis and Haemostasis/Scientific and Standardization Committee Bleeding Assessment Tool (ISTH-SSC BAT) score was 0. Bone age was assessed as 11 years and 4 months using the China-05 scoring method. Brain MRI confirmed a right-sided cochlear implant (Figure 1). Pelvic ultrasonography revealed an infantile uterus. Bilateral breast ultrasound demonstrated breast anlage (9 × 6 mm, 9 × 7 mm), consistent with Tanner stage B2.

To investigate the genetic basis of the patient's short stature and developmental deformities, chromosomal karyotyping and whole-exome sequencing (WES) were performed. Chromosomal karyotype revealed 46, XX. A missense variant in *CDC42* (c. 203G > A, p. Arg68Gln; NM_001791. 4, GRCh38) and two heterozygous variants in *GJB2* (c. 235del, p. Leu79Cysfs*3; c. 109G > A, p. Val37Ile; NM_004004. 6, GRCh38) were identified.

## Treatment and follow-up

**Figure 2:**
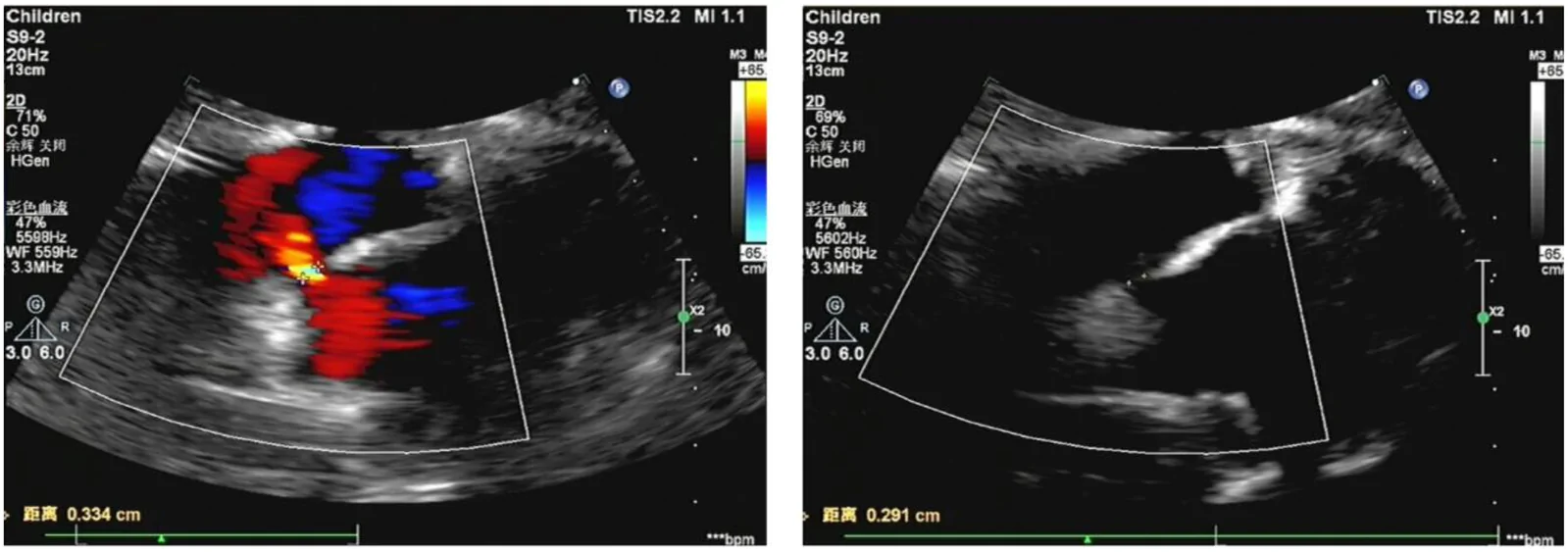
Echocardiographic image of the patient.

As the patient and her parents strongly desired pharmacological intervention for severe short stature, we fully informed the potential risks associated with rhGH therapy, after which we completed the following pre-treatment examinations. Cardiac ultrasonography revealed atrial septal defect, patent foramen ovale, and mild aortic and tricuspid regurgitation (Figure 2). ECG showed sinus arrhythmia. Spinal radiography demonstrated mild lumbar dextroscoliosis. Liver function tests revealed mildly elevated liver enzymes (Table 1). Other laboratory tests (including serum biochemistry, cortisol, adrenocorticotropic hormone, tumor markers, fasting insulin, fasting blood glucose, infectious disease screening, thyroid function, and urinalysis) and ultrasonography of other organ systems revealed no remarkable abnormalities. After multidisciplinary evaluation and comprehensive risk-benefit assessment, and written informed consent obtained from the patient's parents, rhGH was initiated at a dose of 0. 125 U/kg/day.

**Table 1:** Hematological and liver function parameters of the patient.

Laboratory tests	Reference range	Before treatment	6 months post-treatment
WBC (×10⁹/L)	4.3–11.3	4.83	3.97
HGB (g/L)	118–156	132	127
PLT (×10⁹/L)	167–453	118	87
P-LCR (%)	13.0–43.0	43.7	43.1
MPV (fl)	9.4–12.5	12.1	11.2
AST (U/L)	14–44	31	52.1
ALT (U/L)	7–30	35	77.9
GGT (U/L)	5–19	23	23

After one month of treatment, no significant increase in height was observed, though repeated testing revealed that the IGF-1 level rose markedly to 376. 6 ng/mL (−0. 3 SD). Therefore, the rhGH dosage was increased to 0. 15 U/kg/d.

At the 3-month follow-up, the patient's height was 128. 5 cm (−4. 21 SD). The repeated IGF-1 level was 309. 4 ng/mL (−0. 9 SD). After six months of therapy, the height reached 131. 4 cm (−3. 95 SD). Routine blood examination revealed a white blood cell count of 3. 97 × 10⁹/L (4. 3–11. 3 × 10⁹/L) and a platelet count of 87 × 10⁹/L (167–453 × 10⁹/L), accompanied by a slightly elevated large platelet ratio. Occasional giant platelets were seen on peripheral blood smear, and the ISTH-SSC BAT score remained 0. Liver enzyme concentrations were mildly elevated compared with baseline values. The high-sensitivity thyroid-stimulating hormone (hs-TSH) was 6. 464 mIU/L (0. 560–5. 910 mIU/L), whereas free triiodothyronine (FT3) and free thyroxine (FT4) were unremarkable. Bone age reassessment demonstrated no substantial progression. Renal function, fasting blood glucose and fasting insulin showed no significant abnormalities. Given the known impacts of TKS on immune function, we performed a comprehensive immunological evaluation, which demonstrated reduced serum IgG, IgM and IgA concentrations and a low absolute B-lymphocyte count (Table 2). Accordingly, oral levothyroxine was initiated and we advised the patient to avoid crowded environments to lower infection risk. Regular monitoring of height growth was arranged, and close surveillance for cutaneous, mucosal, or other occult bleeding manifestations was recommended.

**Table 2:** Immunological parameters of the patient.

Immunological tests	Reference range	Results
IgA (g/L)	1.0–4.2	0.56
IgG (g/L)	8.6–17.4	5.1
IgM (g/L)	0.5–2.8	0.26
Total IgE (IU/L)	<200	5.0
B lymphocytes (×10⁹/L)	0.09–0.66	0.06
CD4+ T lymphocytes (×10⁹/L)	0.41–1.59	0.66
CD8+ T lymphocytes (×10⁹/L)	0.19–1.14	0.52
NK Cells (%)	7.0–40.0	13.2
Th Cells (%)	27.0–51.0	43.7
Ts Cells (%)	15.0–44.0	34.6
Total T lymphocytes (×10⁹/L)	0.69–2.54	1.23

## Discussion

TKS, first described in 2015, is an ultra-rare monogenic disorder caused by pathogenic variants in the *CDC42* gene. Pathogenic *CDC42* variants alter the amino acid sequence and tertiary structure of the *CDC42* protein, disrupt the physiological GTP-GDP cycling balance, and impair binding affinity with downstream effectors. These molecular aberrations further interfere with intracellular signal transduction, perturb normal embryonic organogenesis and tissue development, and ultimately lead to the multifaceted clinical phenotypes of TKS (1, 8). Based on the localization of variants in functional domains, altered molecular mechanisms and variable clinical phenotypes, *CDC42* pathogenic variants are classified into three distinct subtypes. Type I variants (p. Tyr64Cys, p. Arg66Gly, p. Arg68Gln) are located in the switch II domain of *CDC42*, which compromises GTPase catalytic activity and downstream signal transduction, predominantly causing platelet dysfunction and thrombocytopenia (5, 9, 10). Type II variants occur at or adjacent to the nucleotide-binding pocket (p. Ala159Val, p. Cys81Tyr, p. Ser83Pro), resulting in excessive and constitutive GTPase activation. This abnormal activation disrupts the proliferation, survival, and cytoskeletal dynamics of neural crest-derived cells, leading to severe and typical craniofacial malformations (5, 11–13). Type III variants (p. Ile21Thr, p. Tyr23Cys, p. Glu171Lys) affect key residues mediating the interaction between *CDC42* and downstream effectors harboring CRIB motifs, and their clinical phenotypes closely resemble Noonan syndrome (5, 8, 14).

The present study comprehensively characterizes the clinical and molecular genetic features of a pediatric patient with TKS caused by the rare missense variant *CDC42* c. 203G > A (p. Arg68Gln). To date, only three cases harboring the *CDC42* p. Arg68Gln variant have been reported globally (5, 15), and the detailed clinical profiles and therapeutic strategies of these patients are summarized in Table 3. Cross-case comparative analysis demonstrated high phenotypic consistency among all reported p. Arg68Gln variant carriers, including persistent thrombocytopenia, typical craniofacial and somatic dysmorphisms, short stature and SNHL. The high phenotypic consistency among affected individuals aligns with canonical TKS manifestations, demonstrating that the *CDC42* p. Arg68Gln variant may confer a genetic predisposition to the TKS phenotype.

**Table 3:** Clinical manifestations and treatment of individuals of *CDC42* p.Arg68Gln variant.

Reference	Patients	Hematological manifestations	Neurological manifestations	Growth and development	Other manifestations	Dysmorphic features	Therapy
Case 1 Martinelli et al. (5)	Male, final assessment at age 4	Thrombocytopenia	Sensorineural hearing loss, cognitive deficit Brain MRI: mildly prominent periventricular white matter signal intensities (predominantly posteriorly), ventriculomegaly and increased extra-axial space	Short stature: 4 years old: weight 13 Kg (<3rd percentile, −2.4 SD), height 93 cm (<3rd percentile, −2.29 SD) and occipitofrontal circumference 49 cm (<3rd percentile)	Hypertrophic cardiomyopathy, congenital pulmonary emphysema, OSAS, recurrent maculopapular rash, gastrointestinal bleeding, periodic fever, polyserositis and splenomegaly	Sparse hair with whorls, prominent forehead, epicanthal folds, wide nasal bridge, flared nostrils with a broad nasal tip, long philtrum, an under developed midface, thick ears and a webbed appearance of the neck	Not mentioned
Case 2 Martinelli et al. (5)	Female, final assessment at age 5	Early-onset thrombocytopenia (platelet count 19,000–69,000/mmc), occasional giant platelets identified by blood smear analysis, macrocytic anemia	Sensorineural hearing loss Brain MRI: partial agenesis of the corpus callosum and sub-ependymal heterotopia	Global developmental delay, short stature: 5 years old: weight 15.5 Kg (3–10th percentile), height 101 cm (3–10th percentile)	Ventricular septal defect	Spare hair and eyebrows, broad and prominent forehead, wide nasal bridge, long philtrum, underdeveloped mid-face, cubid's bow, low-set ears	Not mentioned
Case 3 Stubbs et al. (15)	Male, 17 years old	Chronic macrothrombocytopenia	Asymptomatic external hydrocephalus, bilateral sensorineural hearing loss	Global developmental delay, short stature	Recurrent inguinal hernia, OSAS, congenital lobar emphysema (left lower lobectomy was performed)	Biparietal prominence, high forehead, upslanting palpebral fissures, low-set ears with thick helices, a long and smooth philtrum, a thin upper lip, midface hypoplasia, and mandibular prognathia. limited abduction of the shoulders, limited extension of the elbows, camptodactyly of the 5th digits, decreased rotation of the hips, severe thoracic dextroscoliosis and a leg length discrepancy	Corticosteroid
Case 4 Current case	Female, 11 years old	Thrombocytopenia (platelet count 87 × 10⁹/L), occasional giant platelets identified by blood smear analysis, leukopenia (white blood cell count 3.97 × 10⁹/L)	Sensorineural hearing loss, weakened academic performance Brain MRI was limited due to severe artifacts from the cochlear implant	Global developmental delay, short stature: 11 years old: height 123.7 cm (<3rd percentile, −4.35 SD)	Congenital atrial septal defect, hypogammaglobulinemia, humoral immunodeficiency phenotype	Hypertelorism, depressed nasal bridge, widened nasal alar, high arched palate, micrognathia, malocclusion, thick lips, lowset ears, slender fingers, camptodactyly, mild right lumbar scoliosis	Short-acting growth hormone

Macrothrombocytopenia is a hallmark hematological manifestation of *CDC42* switch II domain missense variants, which is observed in most of the patients in Table 3. Of note, macrothrombocytopenia associated with TKS can exhibit an intermittent course, with giant platelets detectable only at discrete time points (16). Mechanistically, previous functional studies have revealed that switch II domain variants induce constitutive overactivation of *CDC42*, which disrupts microtubule and actin cytoskeleton homeostasis, impairs proplatelet maturation, and ultimately reduces platelet production while increasing platelet volume (17, 18). Accordingly, thrombocytopenia with occasional giant platelets observed in this patient is consistent with the intermittent phenotype of macrothrombocytopenia associated with this specific variant. Given the undefined bleeding risk threshold for thrombocytopenia in TKS and the absence of unified clinical management guidelines, long-term dynamic monitoring of platelet count and hemoglobin levels, together with close surveillance for occult bleeding is strongly recommended for TKS patients.

Notably, the patient exhibited leukopenia, reduced serum immunoglobulins and a low B-lymphocyte count, revealing hypogammaglobulinemia and a humoral immunodeficiency phenotype, which may confer increased susceptibility to infections. Rare severe complications, including hydrocephalus, congenital lobar emphysema, necrotizing enterocolitis, pancytopenia, and hemophagocytic lymphohistiocytosis (HLH) -like manifestations, have been previously documented in p. Arg68Gln carriers (15). These atypical phenotypes imply that *CDC42* dysfunction may disrupt neuroepithelial and pulmonary mesenchymal development and trigger excessive immune-inflammatory activation (5). However, due to the extremely limited case number of *CDC42* p. Arg68Gln variants, it remains unclear whether these rare complications represent unique phenotypic characteristics of the p. Arg68Gln variant or sporadic heterogeneous manifestations. Therefore, lifelong, standardized, and systematic monitoring of hematological, immunological, and respiratory systems is essential for precise individualized management of such patients.

Beyond the pathogenic *CDC42* variant, the patient concurrently carried compound heterozygous *GJB2* variants (c. 235del, p. Leu79Cysfs*3; c. 109G > A, p. Val37Ile). The severity of hearing loss in the c. 235del/c. 109G > A compound heterozygous genotype confers only mild impairment (19). However, the patient developed profound SNHL at the age of 4 years and received cochlear implantation, creating an obvious phenotypic discrepancy. This inconsistency suggests that *GJB2* variants were not the dominant driver of the severe auditory phenotype in this case. Accumulated evidence demonstrates that *CDC42* dysfunction disrupts GDP-GTP cycling, leading to SNHL in 84. 6% of TKS patients (1), with all reported p. Arg68Gln carriers exhibiting moderate-to-severe hearing loss. Notably, all reported individuals with the *CDC42* p. Arg68Gln variant present moderate-to-severe SNHL. Accordingly, the patient's profound hearing loss may be predominantly mediated by the pathogenic *CDC42* variant, although a potential aggravating effect of concomitant *GJB2* variants cannot be entirely excluded. Nevertheless, detailed audiometric data in TKS patients remains scarce, warranting further investigation.

**Figure 3:**
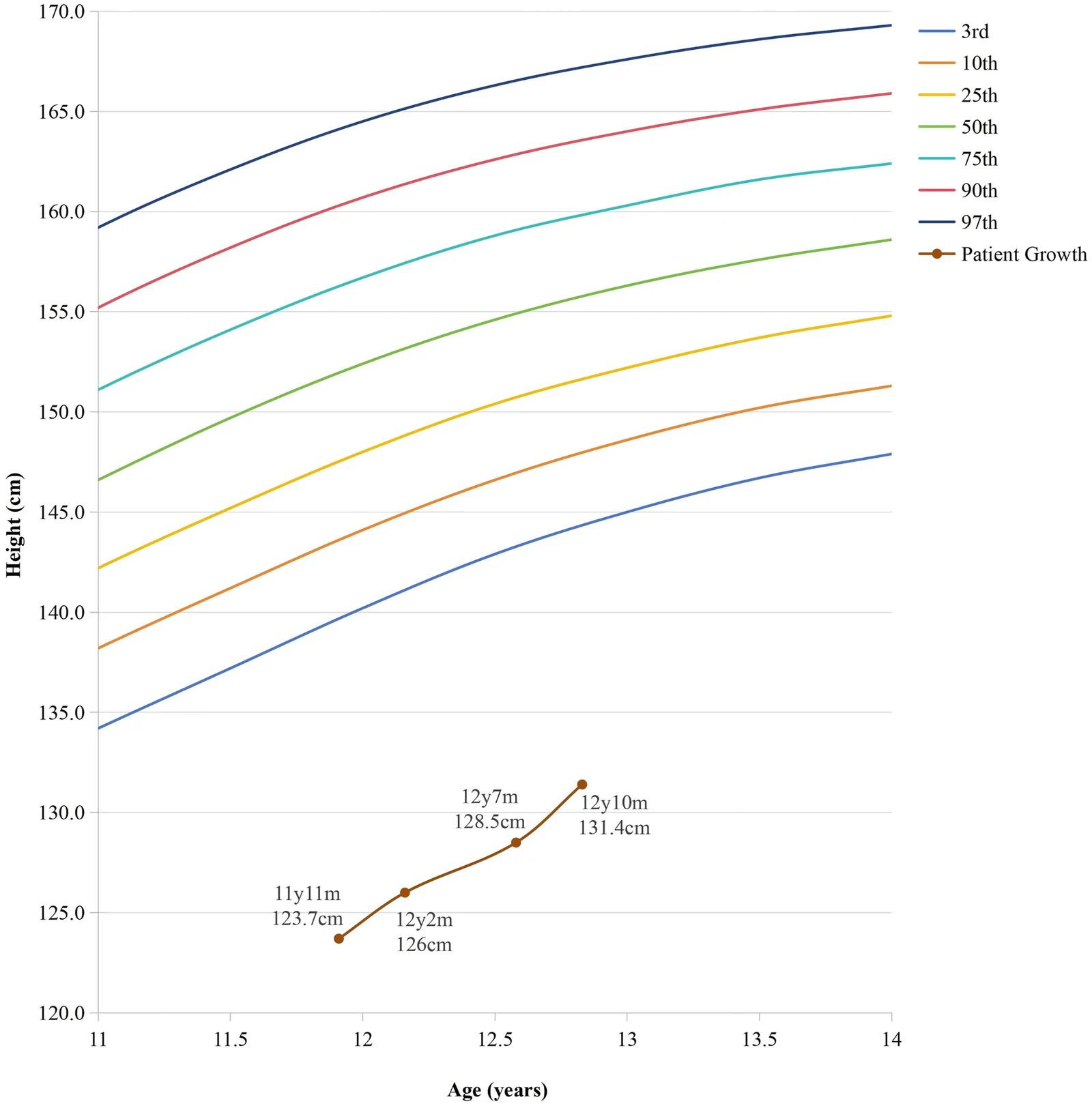
Height growth curve of the patient.

Severe persistent growth retardation was the most prominent clinical problem in this patient, causing significant psychological burden. Currently, no standardized interventions are available for TKS-related short stature. Successful rhGH treatment with favorable growth responses and no severe adverse events has been documented in other genetic syndromes associated with short stature, such as Costello syndrome and KBG syndrome (20, 21). These clinical experiences provide supportive evidence for the off-label rhGH application in TKS. After comprehensive risk-benefit assessment and full informed risk disclosure, we cautiously administered off-label rhGH treatment. The patient achieved obvious height catch-up within six months (Figure 3), accompanied by only mild transient elevations of TSH and liver enzymes without other adverse events. This study is the first to explore rhGH intervention for TKS-related short stature, providing preliminary real-world evidence for the feasibility and safety of this off-label strategy. Long-term follow-up is still required to validate sustained efficacy and safety.

## Conclusion

The study reports a novel pediatric case of TKS caused by the ultra-rare *CDC42* p. Arg68Gln variant, which expands the currently limited phenotypic database of this specific variant. Notably, we explored off-label rhGH intervention for refractory short stature in TKS. Short-term follow-up outcomes preliminarily confirm the efficacy of rhGH in improving linear growth in affected children. Nevertheless, the genotype-phenotype correlation and underlying pathogenic mechanisms of the *CDC42* p. Arg68Gln variant remain to be further elucidated by accumulating additional case data and mechanistic investigations. Long-term continuous follow-up is also essential to comprehensively evaluate the sustained efficacy and safety of rhGH treatment, so as to provide reliable clinical evidence for standardized therapeutic strategies for TKS-related growth retardation.

## Limitations

This study has several limitations. First, parental genetic samples of the patient were not collected owing to the family's preference, which prevented the determination of the cis-trans configuration of the variants. Second, clinical photographic images were unavailable due to the parents’ refusal of public disclosure, resulting in a lack of intuitive phenotypic imaging evidence in the present case. Third, the follow-up duration was limited to six months. Long-term data are required to continuously evaluate the sustained efficacy and safety of rhGH therapy. Fourth, GH provocative testing was not performed in this case. Therefore, whether the growth response to rhGH was mediated through correction of GH deficiency or through other mechanisms remains to be determined.

## Data Availability

The datasets presented in this article are not readily available because due to concerns regarding participant/patients anonymity. Requests to access the datasets should be directed to the corresponding author.
